# Nursing Students’ Experiences with Computer Simulation-Based Communication Education

**DOI:** 10.3390/ijerph18063108

**Published:** 2021-03-17

**Authors:** Ujin Lee, Heeseung Choi, Yeseul Jeon

**Affiliations:** 1 College of Nursing, Seoul National University, Seoul 03080, Korea; bg7917@snu.ac.kr (U.L.); qkznl33@snu.ac.kr (Y.J.); 2 Research Institute of Nursing Science, Seoul National University, Seoul 03080, Korea

**Keywords:** therapeutic communication, nursing students, simulation-based education

## Abstract

Simulation-based communication education has improved nursing students’ communication knowledge and skills. However, communication patterns that students commonly exhibit in simulated situations and students’ responses to specific clinical situations have not been systematically examined. The specific aims of the present study were (1) to identify non-therapeutic communication patterns that nursing students exhibit in simulated situations in the computer simulation-based education (ComEd) program, and (2) explore students’ responses to challenging clinical situations. This study used a mixed-method research design and a convenience sampling method to recruit participants. Frequency analysis and a conventional content analysis method were used to analyze answers provided by participants. A total of 66 students from four Korean nursing schools participated in the study. “False reassurance” was found to be the most common non-therapeutic communication pattern used by nursing students. Nursing students had difficulty in clinical situations such as reporting a patient’s condition to a doctor, communicating with a patient and perform basic nursing skills at the same time, and managing conflicts between patients. Technology-based communication simulation programs, which reflect various clinical situations, are considered a new alternative that can supplement the limitations of clinical practicum and improve the quality of nursing education.

## 1. Introduction

Communication is at the heart of nursing. Previous studies have shown that patient-centered communication is associated with high-quality healthcare, and patients perceived that they received high-quality care when they were listened to by healthcare providers [1,2]. However, performing effective and empathic communication with patients and colleagues in dynamic and case-specific clinical situations is not easy. Nurses may feel challenged when communicating with emotionally charged patients or patients of different sexes, ages, and sociocultural backgrounds when managing the triangle of nurse–physician–patient communication during a conflict [3,4,5,6]. Nursing students reported that they felt uncomfortable, particularly when interacting with patients with acute psychiatric symptoms and when unable to have “meaningful” or “sufficient” communication with patients [4,7].

In spite of the importance of improving communication skills among nursing students, opportunities for having one-on-one communication with patients have decreased due to the combination of rapid increases in numbers of nursing students and difficulties in finding hospital placements for their clinical training [8,9].

Simulation-based education programs have been offered to improve communication competencies among nursing students [10,11,12]. The programs had a significant effect on improving student learning outcomes such as knowledge acquisition and clinical performance. In particular, simulation-based education using standardized patients has been widely used as an alternative for clinical placement, providing hands-on exercises to improve communication skills among students [4,13,14,15]. However, nursing schools with insufficient resources have not been able to offer simulation-based learning experiences for their students due to the difficulties in maintaining the quality and consistency of standardized patients [16].

To compensate for the aforementioned challenges in nursing education, the computer simulation-based interactive communication education (ComEd) program was developed. The main purpose of the ComEd program is to improve communication knowledge, learning self-efficacy, and communication efficacy among nursing students [17]. The program is unique in that it offers nurse–patient communication experiences at a low cost while maintaining the fidelity of the education. The interactive ComEd program was designed to prepare nursing students for interacting with patients with psychiatric symptoms, particularly in challenging clinical situations that require nursing students’ proactive and critical judgment. Challenging situations include notifying the doctor about changes in the patient’s condition, administering medication, and managing conflicts between patients.

Psychiatric nursing didactic classes have emphasized differentiating therapeutic/non-therapeutic communication skills and simulation-based education aimed at evaluating clinical performance and identifying correct/incorrect responses to simulated situations. However, students’ responses to specific situations and non-therapeutic communication patterns that students commonly exhibit in simulated situations have not been systematically examined. In addition, how situations influence students’ learning processes and experiences during a simulation have not been clarified. Understanding nursing students’ communication patterns and learning experiences in simulated situations is important for developing communication education programs.

The theoretical model that guided the present study was Kolb’s Experiential Learning Model [18]. According to Kolb’s theory, effective learning occurs when learners go through a four-stage learning cycle: encountering new experiences (concrete experience), reflecting on the learning experience (reflective observation), analyzing, forming, and modifying ideas from experience (abstract conceptualization), and planning and applying new knowledge (active experimentation). Through the series of interactive clinical performances with virtual patients and a doctor, tailored debriefing sessions with model videos, and reviews of one’s own virtual clinical performance, the participants of the ComEd program are able to experience the four-stage learning cycle.

The specific aims of the present study were (1) to identify non-therapeutic communication patterns that nursing students exhibit in simulated situations and (2) to explore students’ responses in challenging clinical situations. Ultimately, we aimed to establish evidence for improving simulation-based communication education to address nursing students’ needs.

## 2. Materials and Methods

### 2.1. Study Design

The present study used a mixed-method research design to analyze the communication patterns exhibited by nursing students participating in the ComEd program.

### 2.2. Description of ComEd Program

The ComEd program consisted of three parts: a patient medical history video, an interactive clinical performance with two virtual patients (i.e., a depressed patient and a psychotic patient), and a debriefing session that provides tailored feedback based on the answers given by the program user. After logging in, the user first watches the patient’s medical history video and starts interacting with a virtual patient in various clinical situations. During the interaction, the user chooses a response he/she believes appropriate for the situation among multiple-choice questions and records it with their voice or provides an appropriate response for the open-ended questions. Finally, during the debriefing session, the user watches model videos demonstrating therapeutic communication skills and reviews their virtual clinical performance. The program proceeds if the user chooses an appropriate response using therapeutic communication skills (a total of 13 nurse–patient interactions for each scenario); the program is terminated if the user chooses an inappropriate response using non-therapeutic communication skills. The effects of the ComEd program on improving nursing students’ knowledge, communication efficacy, and learning self-efficacy have been demonstrated [17].

### 2.3. Participants and Study Procedure

A convenience sampling method was used to select participants for the present study. Nursing students who (1) had finished a communication class but had not completed a clinical practice in psychiatric wards; and (2) were able to use tablet PCs were eligible to participate in the study. From March to May 2019, we recruited 66 nursing students from four different Korean nursing schools to participate in the ComEd program.

On the study day, researchers explained the study purpose and procedure and obtained informed consent from each participant. Participants were individually provided with a tablet PC with the ComEd program installed and then moved to a quiet and private place to start the program. The ComEd program lasted approximately 40 to 50 min. We offered participants about $20 for their participation.

### 2.4. Data Collection and Analysis

Two types of data were collected while participants interacted with the virtual patients and doctor: (1) the answers selected from multiple-choice questions, and (2) recorded answers participants provided for three challenging situations. The number of multiple-choice questions ranged from 7 to 13, depending on the responses that students chose. Namely, nursing students who chose appropriate responses using therapeutic communication skills in all simulation situations experienced all 13 questions. On the other hand, students who chose inappropriate responses using non-therapeutic communication skills answered at least 7 questions, depending on where they went therapeutically astray. The open-ended questions associated with the three challenging situations included “please report the patient’s condition to the doctor”, “please administer the prescribed medication to the patient”, and “please manage the conflict between the two patients”.

To analyze the multiple-choice answer data, participant answers were first classified into two categories: therapeutic and non-therapeutic responses. Next, we identified the specific communication type of each response (i.e., reflecting, false reassurance) and performed a frequency analysis. Descriptive statistics were used to describe the features of demographic data and multiple-choice answer data.

We used a conventional content analysis method [19] to analyze recording data obtained from answers provided by participants in response to open-ended questions in challenging situations. First, we transcribed audio-recorded data, and the accuracy of the transcripts was confirmed by two authors. Second, two authors independently and repeatedly read the transcripts, identified keywords and phrases, developed a list of codes, and discussed the differences in the coding in research team meetings. Next, based on the three challenging situations, the authors created a matrix and identified main themes [20,21]. Finally, quotes written in Korean were translated into English by a bilingual and bicultural translator, and an independent bilingual reviewer checked the accuracy and quality of the translation.

### 2.5. Ethical Considerations

The study was reviewed and approved by the Institutional Review Board of S. University. Ethical principles were followed for this study. Nursing students voluntarily participated in the study. Informed consent was obtained, and the anonymity of the study was respected.

## 3. Results

Data collected from 66 participants were analyzed in this study. The participants had a mean age of 22.9 (SD ± 1.58) years, and most participants (90.9%) were women. The percentage of men in this sample was slightly lower than the percentage of male nurses (14.7%) in all test takers of the Korean nursing licensing examination in 2020 [22]. More than half (51.5%) had previously received simulation education.

### 3.1. Non-Therapeutic Communication Patterns Nursing Students Exhibited

**Figure 1:**
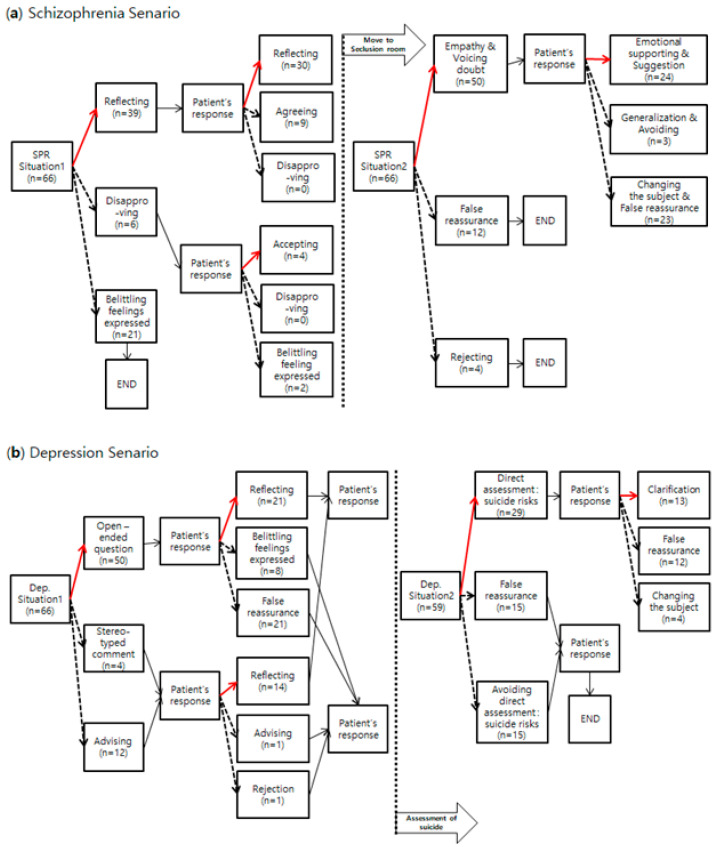
Red lines: the path for therapeutic communication skills; dotted lines: the path for non-therapeutic communication skills. n: number of students who chose the answer. (**a**) Schizophrenia scenario; (**b**) depression scenario.

In both cases, non-therapeutic communication skills frequently used by the participants included “false reassurance” and “expressing a disregarding emotion or belittling feeling”. Participants tended to ignore the needs of the virtual patients without providing explanations or related information. In particular, when communicating with patients experiencing auditory hallucinations and delusions, about 35% (*n* = 23) of participants tried to change the subject and temporarily reassured the patient without managing the patient’s symptoms or providing emotional support. Furthermore, more than 25% (*n* = 15) of the nursing students avoided direct assessment of suicide risk when communicating with a depressed patient who had previously attempted suicide. The contents of the scenarios according to the program’s algorithm and the frequencies of responses selected by the students in each scenario are shown in Figure 1.

### 3.2. Communication Patterns in Challenging Clinical Situations

Participants in the ComEd program had opportunities to interact with virtual patients and doctors in three challenging clinical situations. The themes identified from each challenging situation are summarized in Table 1.

#### 3.2.1. Reporting Changes in a Patient’s Condition to a Doctor

##### Theme 1. Providing Inadequate Patient Information

Even though key elements such as situation, background, assessment, and recommendation are required for clear and effective communication with a doctor, several participants failed to include the key information. For example, they only reported the patient had been disturbing other patients’ sleep without accurately mentioning the patient’s delusions and hallucinations. Furthermore, they omitted key information that the patient’s anxiety and insomnia symptoms did not respond to prescribed medications.

Although nurses play a pivotal communication role with physicians by presenting their opinions about patient treatment and care, only four out of 66 participants presented their opinions about the patient’s state and care.

*Patient Byun-wha Kim, 21-year old, female, is keeping other patients from sleeping. She took the prescribed medication an hour ago.* (ID: Ig12: Not mentioning psychotic symptoms)

*Hello, this is psychiatric nurse, Lee OO. Patient Byun-wha Kim, diagnosed with schizophrenia, is currently anxious, not able to sleep, and disturbing other patients. Her major symptoms are auditory hallucinations and delusions.* (ID: Ij03: Not mentioning about the prescribed medication)

##### Theme 2. Using Ambiguous and Uncertain Expressions

When reporting to a doctor, nursing students often exhibited unclear communication patterns, such as using ambiguous and uncertain expressions. They also hesitated to state, “well…uh…”

*I guess… Patient Byun-wha Kim… seems to have schizo… delusion…* (ID: Ij14)

*Uh…* *Patient… well… patient is hospitalized. Um… chief complaint is uh….*

*… auditory hallucination and delusion…* (ID: Ij05)

#### 3.2.2. Administration of Benzodiazepine

##### Theme 1. Non-Adherence to Medication Administration Guidelines

In the course of administering a prescribed benzodiazepine, more than half of the participants did not adhere to the five rights of medication administration. They mentioned the wrong dosage or route of the medication administration. They failed to explain or confirm the purpose of administering the medication and the adverse side effects that the patient may experience after taking the medication.

*I will give you Ativan 2 mg orally.* (ID: Iy15: Mentioning wrong dosage)

*I will inject Ativan 1 mg. If you continue to feel anxious and are not able to sleep, please let me know.* (ID: Is12: Mentioning wrong route)

##### Theme 2. Lack of Professionalism and Self-Confidence

Nursing students lacked professionalism because of a lack of self-confidence. They often exhibited somewhat daunted and timid attitudes when administering medication.

*I’m just giving you this medication because the doctor said so.* (ID: Is04)

*Uh… the doctor prescribed this medication… um… to help decrease your anxiety… well… Would you like to take this?* (ID: Ig04)

##### Theme 3. Providing False or Vague Reassurance

In addition, nursing students tended to use non-therapeutic communication skills such as providing false or vague reassurance rather than showing empathy or helping patients to find solutions.

*If you take this medication, your hallucination will cease and you would feel comfortable soon.* (ID: Is02)

*I’ll give you the medication. You’ll get better soon and you’ll be discharged.* (ID: Ij09)

*Don’t worry too much. Please take the medication and go to bed with an easy mind.* (ID: Ig15)

*Since you took the medication, your anxiety won’t be bothering you for much longer. Trust me.* (ID: Iy16)

#### 3.2.3. Managing Conflicts between Patients

##### Theme 1. Reluctance to Manage Conflict Situations

The participants of the study tended to avoid direct involvement in the conflict situation or disregard situations when a patient complained of sleep disturbance or conflicts with another patient. They were reluctant to manage the conflicts and tried to terminate the situation.

*The patient has calmed down. I’ve done all that I can do. Please try to get some sleep.* (ID: Is03)

*There’s nothing more I can do because this is a shared room.* (ID: Is14)

*Please remember your hard times (when you were in acute stage) and just try to understand her (another patient’s) situation.* (ID: Iy11)

##### Theme 2. Lack of Empathy and Attentive Listening

On the other hand, some students focused on conflict resolution without expressing empathy or attentive listening.

*I’m sorry. I’ll change your room.* (ID: Iy15)

*I’ll tell your doctor about your discomfort if you want.* (ID: Is05)

In both types of responses, either they avoided involvement in conflict situations or focused on problem-solving; they were not able to respond to a patient’s feelings and needs using therapeutic communication skills.

## 4. Discussion

Previous research has demonstrated the effects of simulation-based education in improving nursing knowledge, communication skills, critical thinking abilities, and situation management abilities [12,16,23,24]. However, we found very little information in the literature concerning how students’ learning processes occur and how students respond in simulated situations. Hence, we identified non-therapeutic communication patterns that students exhibited when interacting with psychiatric patients and nursing students’ responses in three challenging clinical situations through the ComEd program.

### 4.1. Non-Therapeutic Communication Patterns That Nursing Students Commonly Exhibited

“False reassurance” was found to be the most common communication pattern used by nursing students who participated in the study in interactions with both a depressed patient and a psychotic patient. Although nurses or therapists can use reassurance to reduce patient anxiety and manage symptoms, previous studies have shown that reassuring patients is often ineffective because the patient understands or interprets the therapist’s reassuring statements differently than intended [25,26,27]. In other words, temporarily reassuring a patient can be seen as a therapist ignoring or belittling a patient’s problems, and the patient may also feel the therapist does not take his or her problems or feelings seriously. It will also block the patient from gravely exploring their emotions. Consequently, false reassurance may inhibit nurse–patient communication or lead to premature relationship termination [28]. The meaningless conversation continues without a therapeutic process, and the patient’s needs are not met.

Additionally, in situations where the patient directly mentioned their symptoms (e.g., complaining of delusions or hallucinations or expressing suicidal ideas), many participants abruptly changed the conversation or showed a defensive attitude without further assessment or intervention. This finding is different from that of previous studies with nurses who had 5 to 25 years of psychiatric ward experience [29,30]. In those studies, nurses actively formed therapeutic relationships with schizophrenic patients and patients with depression. The findings showed that clinical experiences communicating with patients are important for performing an effective and proactive intervention of patients’ symptoms; in fact, prior studies explained that nursing students felt a great burden and complained about anxiety related to communicating or interacting with psychiatric patients [31,32]. Thus, for improving students’ communication competencies, repetitive exposures and practices with different types of psychiatric cases are needed.

In particular, when communicating with depressed patients with suicidal ideas, participants showed non-therapeutic communication, such as avoiding direct assessment or the situation altogether, despite needing to assess suicidal thoughts and plans that could affect patient safety. Nurses’ tendency to avoid sensitive topics such as suicide has been observed in previous studies [33,34]. Nurses were silent because they did not know what to say to a suicidal patient or because of the lack of suicide assessment training; they ignored the problem because they were afraid that the patient would be harmed [33,34]. Therefore, more simulation education using various scenarios is needed to improve nursing students’ therapeutic communication skills and abilities to interact with patients and deal with sensitive topics. The ComEd program, which allows nursing students to repeatedly practice scenarios, could be an effective approach.

### 4.2. Communication Patterns in Challenging Clinical Situations

Our study illustrated communication patterns in three challenging clinical situations. First, the majority of study participants exhibited various difficulties reporting changes in a patient’s condition to a doctor, such as missing key information, using ambiguous words, repeating the same words with hesitation, and failing to make suggestions to the doctor regarding a patient treatment plan. This finding was consistent with the results of previous studies that showed inexperienced nursing students complained of considerable difficulty and communicated inadequately and unclearly in a series of situations where they must judge and analyze patients’ problems based on theoretical knowledge and communicate with other medical staff [35]. Kim et al. [36] also reported that nurses were reluctant to make suggestions to doctors because they felt awkward and uncomfortable. This attitude could be the result of the hierarchical culture of hospitals, particularly in Korean society.

The importance of communication between medical staff members, including doctor-to-nurses or nurse-to-nurse, is being emphasized [37,38]. Inefficient communication between medical staff members is a key issue that causes medical accidents and delays in treatment, which threatens patient safety. In particular, the omission of patient information was the most serious problem [39,40]. Nevertheless, communication education provided by most nursing colleges is limited to communication between patients and nurses [41], and there is also no systematic education on reporting or handing over duties [42]. Therefore, a program that allows nursing students to experience and practice communication between medical staff in various scenarios is necessary.

The Joint Commission International (JCI) proposed situation, background, assessment, and recommendation (SBAR), which is a standardized communication tool for effective communication between medical staff members and has been used in various medical institutions, leading to increased interest in structured frameworks in nursing education [35,43]. SBAR communication facilitates communication between medical staff members and improves collaborative relationships among them [44]. This study is meaningful in that the nursing students were able to experience assessing and reporting patients’ status to doctors and learn an effective communication method using SBAR through communication simulation and debriefing sessions in the ComEd program.

Second, the majority of participants noted that they did not properly follow the five rights in the course of administering prescribed medication and did not explain the purpose of the drug administration, the patient’s reactions, and side effects that could occur. Even participants who explained the necessity of drug administration showed a lack of expertise with a passive and insecure attitude. Moreover, participants still used non-therapeutic communication after drug administration, such as vague expectations and false reassurance, rather than emotional support or empathy with patients. These results revealed that participants made mistakes because they felt burdened or embarrassed when they had to communicate with a patient and perform basic nursing skills at the same time.

In a clinical environment, nurses are required to perform various tasks simultaneously, but most nursing colleges provide communication education separately. This study showed that nursing students were challenged with clinical situations requiring an integrated nursing performance. Therefore, it is necessary to develop a variety of scenarios with integrated situations that include both nursing and communication skills in various clinical situations.

Third, participants managing conflicts between the patients tended to focus on solution-oriented communication rather than listening to and empathizing with the patient’s complaints. Studies have shown that empathy is one of the most important factors of patient-centered communication [45], and nurses’ empathic skills affect patients’ health outcomes [46]. Simulation has become an effective tool for acquiring empathic communication skills [47], and immediate feedback from educators is a critical component of the learning process [48]. The voice recording feature and tailored individual debriefing sessions of the ComEd program could improve nursing students’ empathic skills.

The present study has several limitations. First, nursing students’ communication experiences were evaluated only in a simulated environment. Therefore, future research must investigate nursing students’ use of communication skills in an actual clinical setting. Second, we were able to collect verbal communication data only; thus, we were not able to explore students’ non-verbal communication patterns, which are an integral component in patient care [49]. Lastly, this study involved 66 nursing students recruited from four universities, and male nursing students were underrepresented in the sample. This restricts the generalizability of our results.

### 4.3. Directions of Communication Education in Nursing

The importance of clinical education is emphasized in maintaining the quality of nursing. However, as the number of nursing students rapidly increases [9], clinical placement opportunities in special wards such as psychiatric wards have decreased, limiting students’ opportunities to communicate one-on-one with patients who have psychiatric symptoms. The opportunity for nursing students’ direct and active involvement in patient care is also decreasing due to changes in the clinical placement environment, including recent changes in awareness of patients’ rights and enhanced measures to protect personal information [50,51].

Using the technology-based interactive communication simulation education program ComEd, the present study provided nursing students with opportunities to interact with virtual patients and doctors in various situations. The findings of the study demonstrated the need for a nursing simulation that offers nursing students opportunities to practice communication skills in challenging situations. Particularly, scenarios where students can practice and learn how to differentiate between false reassurance and emotional support, how to communicate with doctors with confidence, and how to interact with challenging patients are needed. In addition, simulation scenarios integrating nursing skills and communication skills could help students acquire critical thinking abilities and integrated nursing skills.

Moreover, evaluation methods that consider communication education efficacy and effectiveness need to be improved to increase education quality. Currently, the evaluation of communication-related knowledge and skills in most nursing colleges focuses on performance, making it difficult to assess individual characteristics such as learning attitude, level of participation in education, learning speed, and learning depth. As observed in our study, computer simulation-based education can track each step of users’ progress, assess users’ learning activities during the entire process in various ways (voice recording, video recording, etc.), and provide personalized feedback according to the users’ proficiency level.

Nursing education needs to be prepared in the post-COVID-19 era. Many didactic courses in nursing are already being delivered online, and educators, who need to offer clinical practicum in consideration of patient and student safety during the COVID-19 pandemic, have reported cases of online simulation-based practicum sessions [52,53]. It is necessary to establish a foundation for high-quality nursing education appropriate for the post-COVID-19 era through the continuous development of technology-based communication simulation programs that can replicate various clinical scenarios. Technology-based online simulation education makes it easy to evaluate the learning activities of individual students, continuous curriculum evaluation of real situations without time or space constraints and provides personalized feedback [54]. Therefore, the efficiency of education can be improved through learner-centered education. In the future, various types of programs using virtual reality or augmented reality techniques would further allow students to experience a more lifelike clinical practicum.

## 5. Conclusions

In this study, we assessed nursing students’ communication patterns used in clinical settings by allowing nursing students to participate in a computer simulation-based communication program. In addition, we identified the areas on which the communication education program needs to focus and directions for the future program. Technology-based communication simulation programs, which reflect various clinical situations, are considered a new alternative that can supplement the limitations of clinical practicum and improve the quality of nursing education.

Accumulated knowledge obtained from the analysis of communication patterns provides evidence for the development of communication education contents that reflect both nursing educational goals and the students’ needs. In addition, the widespread use of technology-based communication simulation programs will help nursing students build confidence in the clinical setting and improve nursing competency. Furthermore, this study can inform nursing educators on how to maximize the efficiency of communication education.

## Figures and Tables

**Table 1:** Themes emerged in challenging clinical situations.

Challenging Clinical Situation	Themes
Reporting changes in a patient’s condition to a doctor	Providing inadequate patient information
	Using ambiguous and uncertain expressions
Administration of benzodiazepine	Non-adherence to medication administration guidelines
	Lack of professionalism and self-confidence
	Proving false or vague reassurance
Managing conflicts between patients	Reluctance to manage conflict situations
	Lack of empathy and attentive listening

## Data Availability

The data presented in this study are available on request from the corresponding author. The data are not publicly available due to ethical restrictions.

## References

[1] Rutten L.J.F., Aqunwamba A.A., Beckjord E., Hesse B.W., Moser R.P., Arora N.K. (2015). The Relation Between Having a Usual Source of Care and Ratings of Care Quality: Does Patient-Centered Communication Play a Role?. J. Health Commun..

[2] Stefanini A., Aloini D., Gloor P., Pochiero F. (2020). Patient Satisfaction in Emergency Department: Unveiling Complex Interactions by Wearable Sensors. J. Bus. Res..

[3] Ali M. (2018). Communication skills 6: Difficult and Challenging Conversation. Nurs. Times.

[4] Lehr S.T., Kaplan B. (2013). A Mental Health Simulation Experience for Baccalaureate Student Nurses. Clin. Simul. Nurs..

[5] Norouzinia R., Aghabarari M., Shiri M., Karimi M., Samami E. (2015). Communication Barriers Perceived by Nurses and Patients. Glob. J. Health Sci..

[6] Sheldon L.K., Barrett R., Ellington L. (2006). Difficult Communication in Nursing. J. Nurs. Sch..

[7] Wojtowicz B., Hagen B., Van Daalen-Smith C. (2014). No Place to Turn: Nursing Students’ Experiences of Moral Distress in Mental Health Settings. Int. J. Ment. Health Nurs..

[8] Kim H.S. (2013). Problems and Improvement Plans of Practical Education in Health and Nursing. Korean Council for College University Education. Report. http://hver.kcce.or.kr/board/4002000/AA2CD919/170.

[9] Korean Nurses Association (2019). Statistics of Nursing Education Institutions. http://www.koreanurse.or.kr/resources/statistics.php.

[10] Kim J., Heo N., Jeon H.J., Jung D. (2015). Effects of Simulation Education on the Communication Competence, Academic Self-Efficacy, and Attitude About the Elderly for Nursing Student: A Learning Approach Based on an Elderly with Cognition Disorder Scenario. J. Korean Acad..

[11] Zavertnik J.E., Huff T.A., Munro C.L. (2010). Innovative Approach to Teaching Communication Skills to Nursing Students. J. Nurs. Educ..

[12] Sherwood R.J., Francis G. (2018). The Effect of Mannequin Fidelity on the Achievement of Learning Outcomes for Nursing, Midwifery and Allied Healthcare Practitioners: Systematic Review and Meta-Analysis. Nurse Educ. Today.

[13] Choi Y. (2012). Exploring Experiences of Psychiatric Nursing Simulations Using Standardized Patients for Undergraduate Students. Asian Nurs. Res..

[14] Choi H., Hwang B., Kim S., Ko H., Kim S., Kim C. (2016). Clinical Education in Psychiatric Mental Health Nursing: Overcoming Current Challenges. Nurse Educ. Today.

[15] Park S.Y., Kweon Y.R. (2012). The Effect of Using Standardized Patients in Psychiatric Nursing Practical Training for Nursing College Students. J. Korean Acad. Psychiatr. Ment. Health Nurs..

[16] Guise V., Chambers M., Välimäki M. (2012). What Can Virtual Patient Simulation Offer Mental Health Nursing Education?. J. Psychiatr. Ment. Health Nurs..

[17] Choi H., Lee U., Jeon Y.S., Kim C. (2020). Efficacy of the Computer Simulation-Based, Interactive Communication Education Program for Nursing Students. Nurse Educ. Today.

[18] Kolb D.A. (2015). Experiential Learning: Experience as the Source of Learning and Development.

[19] Sandelowski M. (2000). Whatever Happened to Qualitative Description?. Res. Nurs. Health.

[20] Bernard H.R. (2000). Social Research Methods: Qualitative and Quantitative Approaches.

[21] Patton M.Q. (2002). Qualitative Research and Evaluation Methods.

[22] (2020). The Korea Times. Korea Sees Increase in Male Nurses. https://www.koreatimes.co.kr/www/nation/2020/10/119_297611.html.

[23] Cant R.P., Cooper S.J. (2010). Simulation-Based Learning in Nurse Education: Systematic Review. J. Adv. Nurs..

[24] La Cerra C., Dante A., Caponnetto V., Franconi I., Gaxhja E., Petrucci C., Alfes C.M., Lancia I. (2019). Effects of High-Fidelity Simulation Based on Life-Threatening Clinical Condition Scenarios on Learning Outcomes of Undergraduate and Postgraduate Nursing Students: A Systematic Review and Meta-Analysis. BMJ Open.

[25] McDonald I.G., Daly J., Jelinek V.M., Panetta F., Gutman J.M. (1996). Opening Pandora’s Box: The Unpredictability of Reassurance by a Normal Test Result. BMJ.

[26] Petrie K.J., Müller J.T., Schirmbeck F., Donkin L., Broadbent E., Ellis C.J., Gamble G., Rief W. (2007). Effect of Providing Information About Normal Test Results on Patients’ Reassurance: Randomised Controlled Trial. BMJ.

[27] Rief W., Heitmüller A.M., Reisberg K., Rüddel H. (2006). Why Reassurance Fails in Patients with Unexplained Symptoms—An Experimental Investigation of Remembered Probabilities. PLoS Med..

[28] Lee M., Yang S., Kim K., Jung M., Kang R., Park K., Lee J., Jung A., Hyun M. (2019). Psychiatric Mental Health Nursing.

[29] Pounds K.G. (2010). Client-Nurse Interaction with Individuals with Schizophrenia: A Descriptive Pilot Study. Issues Ment. Health Nurs..

[30] Hagen J., Knizek B.L., Hjelmeland H. (2017). Mental Health Nurses’ Experiences of Caring for Suicidal Patients in Psychiatric Wards: An Emotional Endeavor. Arch. Psychiatr. Nurs..

[31] Sarikoc G., Ozcan C.T., Elcin M. (2017). The Impact of Using Standardized Patients in Psychiatric Cases on the Levels of Motivation and Perceived Learning of the Nursing Students. Nurse Educ. Today.

[32] Choi S.H., Byun E.K. (2020). The Lived Experiences of Psychiatric Nursing Practice among Nursing Students. J. Converg. Cult. Technol..

[33] Bolster C., Holliday C., Oneal G., Shaw M. (2015). Suicide Assessment and Nurses: What Dose the Evidence Show?. Online J. Issues Nurs..

[34] Valente S. (2011). Nurses’ Psychosocial Barriers to Suicide Risk Management. Nurs. Res. Pract..

[35] Thomas C.M., Bertram E., Johnson D. (2009). The SBAR Communication Technique: Teaching Nursing Students Professional Communication Skills. Nurse Educ..

[36] Kim H.Y., Jeong Y.J., Kang J., Mun H.S. (2016). The Effect of SBAR Reports on Communication Clarity and Nurse-Physician Collaborative Relationships: A One Group Pretest-Posttest Design. J. Muscle Joint Health.

[37] Berkenstadt H., Haviv Y., Tuval A., Shemesh Y., Megrill A., Perry A., Rubin O., Ziv A. (2008). Improving Handoff Communications in Critical Care: Utilizing Simulation-Based Training Toward Process Improvement in Managing Patient Risk. Chest.

[38] Leonard M.W., Frankel A.S. (2011). Role of Effective Teamwork and Communication in Delivering Safe, High-Quality Care. Mt. Sinai J. Med..

[39] Flemming D., Hübner U. (2013). How to Improve Change of Shift Handovers and Collaborative Grounding and What Role Does the Electronic Patient Record System Play? Results of a Systematic Literature Review. Int. J. Med. Inform..

[40] Lee J.Y. (2015). Effective Communication for Patient Safety. J. Korean Med. Assoc..

[41] Son H.M., Kim H.S., Koh M.H., Yu S.J. (2011). Analysis of the Communication Education in the Undergraduate Nursing Curriculum of Korea. J. Korean Acad. Soc. Nurs. Educ..

[42] Ha Y.K., Lee Y.J., Lee Y.H. (2017). Simulation Training Applying SBAR for the Improvement of Nursing Undergraduate Students’ Interdisciplinary Communication Skills. J. Korean Data Inf. Sci. Soc..

[43] Noh Y.G., Lee I. (2018). Effect of Stepwise Communication Education Program Using SBAR Among Nursing Students: Focusing on Scenarios and Nursing Case-Based Role Playing. J. Korean Acad. Soc. Nurs. Educ..

[44] Woodhall L.J., Vertacnik L., McLaughlin M. (2008). Implementation of the SBAR Communication Technique in a Tertiary Center. J. Emerg. Nurs..

[45] Neumann M., Bensing J., Mercer S., Ernstmann N., Ommen O., Pfaff H. (2009). Analyzing the “Nature” and “Specific Effectiveness” of Clinical Empathy: A Theoretical Overview and Contribution Towards a Theory-Based Research Agenda. Patient Educ. Couns..

[46] Kelm Z., Womer J., Walter J.K., Feudtner C. (2014). Interventions to Cultivate Physician Empathy: A Systematic Review. BMC Med. Educ..

[47] Bauchat J., Park C., Santos J., Anderson L. (2016). Simulation-Based Empathetic Communication Curriculum. MedEdPORTAL.

[48] Furnes M., Kvaal K.S., Høye S. (2018). Communication in Mental Health Nursing-Bachelor Students’ Appraisal of a Blended Learning Training Programme-an Exploratory Study. BMC Nurs..

[49] Ruben M.A., Blanch-Hartigan D., Hall J.A. (2017). Nonverbal Communication as a Pain Reliever: The Impact of Physician Supportive Nonverbal Behavior on Experimentally Induced Pain. Health Commun..

[50] Lim K.C. (2011). Directions of Simulation-Based Learning in Nursing Practice Education: A Systematic Review. J. Korean Acad. Soc. Nurs. Educ..

[51] Kim H.J., Huh J.S. (2013). The Right of the Clinical Training for the Medical Students and Privacy of the Patients. Korean J. Med. Law.

[52] Esposito C.P., Sullivan K. (2020). Maintaining Clinical Continuity Through Virtual Simulation During the COVID-19 Pandemic. J. Nurs. Educ..

[53] Stanley M.J., Serratos J., Matthew W., Fernandez D., Dang M. (2018). Integrating Video Simulation Scenarios into Online Nursing Instruction. J. Nurs. Educ..

[54] Kim H., Yu S. (2008). An Online Learning System for Evaluating Learner’s Activities and Study Level. JKSCI.

